# Complete Suprapatellar Plica in a Handball Player: A Case Report

**DOI:** 10.5704/MOJ.2407.012

**Published:** 2024-07

**Authors:** PM Santos, A Moreira, QJ Costa, J Machado, NC Barbosa

**Affiliations:** Department of Orthopaedic Surgery, Pedro Hispano Hospital, Matosinhos, Portugal

**Keywords:** arthroscopy, knee, suprapatellar plica

## Abstract

We report on a 19-year-old female patient who was diagnosed with a complete suprapatellar plica syndrome. She underwent arthroscopic excision of the plica. Post-operatively, there was complete resolution of the symptoms, with return to sports activity. A complete suprapatellar plica is a rare condition that separates the suprapatellar pouch from the rest of the knee. Cases of symptomatic complete suprapatellar plica should be managed with conservative measures initially. If conservative therapy fails, surgical arthroscopic excision is required.

## Introduction

Knee plicae are anatomic structures remnant of the embryologic synovial membrane. There are four types of synovial knee plicae according to their location: infrapatellar, mediopatellar, suprapatellar, and lateral^1^.

Suprapatellar plica is the most common and divides the suprapatellar pouch from the rest of the knee joint. The importance of suprapatellar plica is still debatable because they are usually normal findings during arthroscopy or cadaveric studies^1^. Cases of complete suprapatellar plica dividing the knee joint into two separate compartments are rare.

We present a rare case of a symptomatic complete suprapatellar plica presenting after direct trauma, successfully treated by resecting the plica arthroscopically. A complete suprapatellar plica should always be considered as a differential diagnosis in cases of prolonged anterior knee pain and suprapatellar swelling.

## Case Report

A 19-year-old handball player presented to our clinic with complaints of discomfort above the patella of the right knee after direct trauma playing handball. The discomfort worsened with prolonged sitting, stair climbing and physical activity.

Clinical examination revealed a painless suprapatellar fluid collection. Range of motion of the right knee was normal, but painful with knee flexion. Patellar grind and apprehension test were both negative. Ligamental and meniscal manoeuvres were negative as well.

Standard anteroposterior and lateral radiographs showed no fractures or bony lesions and were otherwise normal. An initial six-month trial of conservative treatment with resting, non-steroidal anti-inflammatory and physiotherapy followed by a trial of intra-articular corticoid injection were unsuccessful.

**Fig. 1:**
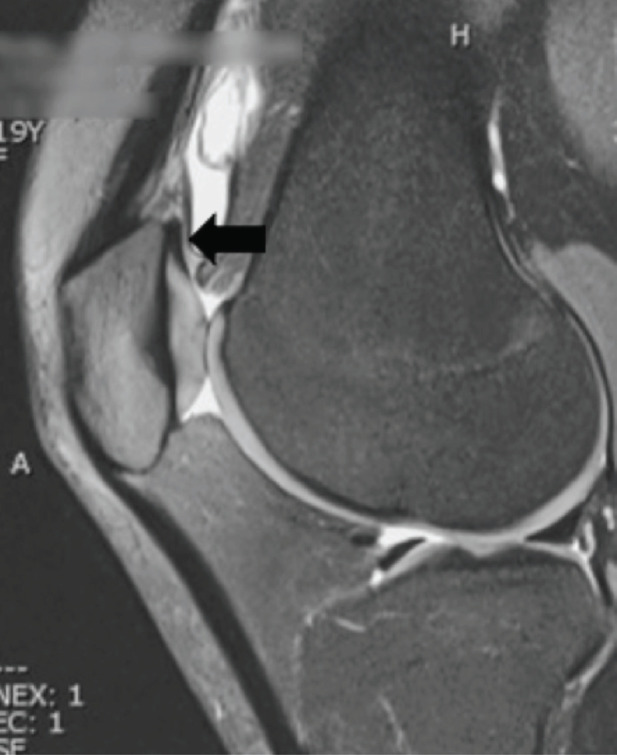
MRI imaging. Complete suprapatellar plica (arrow) preventing communication between the sub quadricipital recess and the rest of the joint space.

Further examination with MRI imaging revealed a complete suprapatellar plica (Type F according to Dandy classification)^2^ preventing communication between the sub quadricipital recess and the rest of the joint space, resulting in an effusion limited to the sub quadricipital recess. There were no other relevant findings on the MRI imaging (Fig. 1).

She underwent arthroscopic removal of the complete plica. There were no intra-operative or post-operative complications. The patient was placed supine on the operative room under spinal anaesthesia and an arthroscopy was performed through anteromedial and anterolateral working portals. We used a standard 30° arthroscope.

**Fig. 2:**
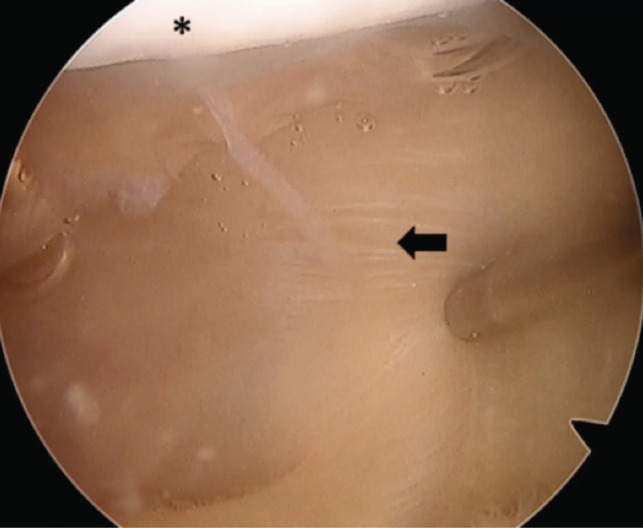
Intra-operative arthroscopic image. View of the complete suprapatellar plica (arrow) proximal to the patella (*).

The arthroscopy confirmed the MRI findings, and the suprapatellar plica was totally excised with a shaver device. We highlight the importance of careful resection to prevent damaging the quadriceps tendon (Fig. 2). Careful identification of the quadriceps tendon during arthroscopy is essential. The procedure was performed with the leg in full extension and during debridement we recommend confirming the quadriceps tendon periodically to avoid quadriceps resection.

**Fig. 3:**
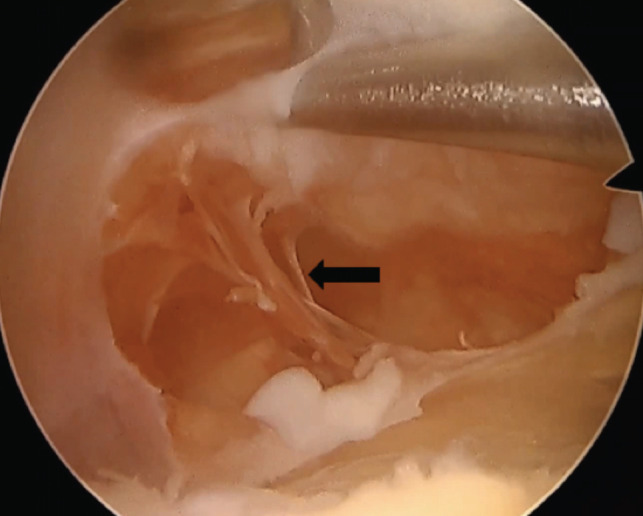
Arthroscopic view after the resection of the suprapatellar plica. The suprapatellar pouch was covered in an inflamed synovium (arrow) distinct of the rest of the knee cavity.

The suprapatellar pouch was covered in an inflamed synovium distinct from the rest of the knee cavity (Fig. 3). Tissue samples were collected, and anatomical pathology analysis revealed fibrous tissue covered by synovium with hyperplasic aspects and the presence of a mononuclear inflammatory infiltrate.

Post-surgery the patient was allowed full weight bearing. She presented a full recovery, with no knee discomfort at the two weeks follow-up consultation. At six months and one year follow-up there was no symptomatic recurrence.

## Discussion

Complete suprapatellar plicae are a rare entity. Dandy *et al* found a 4.2% rate of complete suprapatellar plicae in their study with 500 arthroscopies^2^. Akao *et al* in their study of 125 arthroscopies, all patients diagnosed with suprapatellar plica syndrome were identified with complete septum. According to the literature, it is more likely that pathological changes in the suprapatellar plica occur in the complete type plica^3^. It is extremely important to determine if the plica is the cause of knee symptoms and other intra articular lesions must be ruled out such as meniscus injury, cartilage injury and tendinitis^1^. Our case was a complete type suprapatellar plica with no other intraarticular lesions, successfully treated with arthroscopic excision, thus highlighting the importance of orthopaedic physicians being able to suspect this diagnose when treating patients with suprapatellar pain or swelling.

Literature about complete suprapatellar plicae is scarce. Initially conservative treatment with rest, non-steroidal anti-inflammatory drugs and physiotherapy was tried with our patient. We followed Zmerly *et al* recommendations, if conservative treatment fails after three to six months, arthroscopy should be done both to diagnose and treat^1^.

Arthroscopic excision is the gold standard for surgical treatment. Johnson *et al* have reported an 80% success rate after arthroscopic plica excision compared to only 30% in the control group submitted to diagnostic arthroscopy alone^4^. Bae *et al* reported 34 cases of complete suprapatellar plicae submitted to arthroscopic resection with a success rate of 90% after surgery^5^.

In conclusion, complete suprapatellar plicae are rare but should be considered in patients with anterior knee pain. Conservative treatment is initially recommended. Arthroscopic excision should be performed when conservative therapy fails.
